# Improved YOLOv8 with average pooling downsampling for detection and classification of intertrochanteric femoral fractures in X-ray images: a study focusing on AO/OTA classification

**DOI:** 10.3389/fmed.2026.1759383

**Published:** 2026-03-02

**Authors:** Zheming Shen, Yu Wang, Yu Chen, Haowen Lu, Can Tang, Zhiheng Gao, Xuequan Zhao, Haifu Sun, Yuchen Qian, Youbin Zhang, Yusen Qiao

**Affiliations:** 1 Department of Orthopaedics, The First Affiliated Hospital of Soochow University, Suzhou, China; 2 Xiangcheng District Caohu People’s Hospital, Suzhou, China; 3 Cangzhou Integrated Traditional Chinese and Western Medicine Hospital, Cangzhou, China

**Keywords:** artificial intelligence, hip fracture, intertrochanteric femoral fracture, medical imaging, object detection, YOLOv8

## Abstract

**Objective:**

This study aims to develop an artificial intelligence system for the accurate detection and classification of intertrochanteric femoral fractures (types A1–A3 according to the AO/OTA classification) in X-ray images, focusing on improving precision and optimizing computational efficiency.

**Methods:**

This study adopted a retrospective design, using 976 X-ray image datasets collected from hospital archives. The images were preprocessed, annotated by orthopedic specialists, and divided into training and test sets. The model was improved by replacing the traditional convolutional downsampling modules in YOLOv8 with Average Pooling Downsampling (ADown) modules to enhance feature extraction for small fracture targets. Model training incorporated data augmentation techniques and was evaluated using metrics such as precision, recall, and mean Average Precision (mAP).

**Results:**

The proposed YOLOv8-ADown model achieved an overall mAP50 of 81.7%, higher than the 80.5% of the original YOLOv8. The detection precision for A1, A2, and A3 type fractures increased by 7.3, 3.5, and 7.8%, respectively. Furthermore, the number of model parameters was reduced by 12.3%, and computational complexity (FLOPs) was decreased by 9.8%, demonstrating potential for deployment on edge devices.

**Conclusion:**

The YOLOv8-ADown model provides an efficient solution for fracture detection and is expected to assist in clinical diagnosis. Future work should address data collection challenges and conduct multi-center validation.

## 1 Introduction

Hip fracture is a significant global health problem, especially among the elderly population, where untimely diagnosis can lead to high morbidity and mortality (1, 2). According to the Global Burden of Disease Study, the incidence and prevalence of hip fractures in patients aged 55 and over have continued to rise over the past three decades. In 2019, the global age-standardized incidence rate reached 681.35 per 100,000 people, highlighting its substantial disease burden (3). Accurate diagnosis is crucial for treatment decisions; for instance, early detection can help physicians differentiate between surgical intervention (such as internal fixation or arthroplasty) and conservative treatment, thereby optimizing patient outcomes, reducing complications, and lowering healthcare costs (4, 5). However, traditional radiological diagnosis highly depends on the physician’s level of experience, leading to significant variability in diagnostic sensitivity and specificity. Studies have shown that the sensitivity of fracture detection by general practitioners can be as low as 69.2%, while that of specialists can reach 96.2%. This disparity underscores the necessity for auxiliary tools to reduce diagnostic errors and missed diagnoses (6–8). Furthermore, the clinical utility of AI tools can be amplified by integrating them with biomechanical principles, where optimization of internal fixation configurations and external fixator designs can enhance treatment precision, thereby providing multidisciplinary support for the YOLOv8-ADown model (9, 10).

In recent years, artificial intelligence (AI) has emerged as a promising method for medical image analysis. However, existing models still face challenges of insufficient accuracy and precision when dealing with small targets like fractures (11–13). For example, most AI research focuses on binary classification (fracture vs. non-fracture) (14), with only a few studies incorporating fracture grading standards, such as the Garden classification for femoral neck fractures (15). There is a lack of fine-grained classification of fracture types (e.g., AO/OTA classification), and an inability to provide precise localization of the fracture area (16, 17). Although some work has attempted to integrate interpretability techniques like Grad-CAM, these methods often fail to directly segment fracture lines, limiting their clinical applicability (18). Furthermore, evaluations of deep learning-based decision support systems in real clinical environments have shown limited performance improvement when collaborating with human doctors and a risk of high bias (19).

Addressing these shortcomings, this study proposes an improved framework based on YOLOv8—the YOLOv8-ADown model. By replacing the traditional convolutional downsampling modules with Average Pooling Downsampling (ADown) modules, the feature extraction capability for small fracture targets is optimized. This framework not only achieves fracture detection, and classification and but also significantly improves computational efficiency. Preliminary results indicate that the proposed YOLOv8-ADown framework significantly enhances detection accuracy and computational efficiency, addressing the limitations of existing models in fine-grained fracture classification. Compared to previous studies, the advantages of this framework are: 1. It provides multi-class fracture grading (AO/OTA types A1–A3), enhancing interpretability; 2. Through the combination of attention mechanisms and pooling optimization, it balances accuracy and speed, making it suitable for deployment on edge devices (20, 21).

Looking forward to future applications, this model is expected to serve as a clinical auxiliary tool, integrated into radiological workflows, helping doctors quickly identify subtle fractures, particularly in high-volume or resource-limited scenarios (22). Multi-center validation and real-time deployment will be the next key steps to assess its generalizability and clinical impact (23, 24). Simultaneously, by integrating with osteoporosis screening and risk stratification tools (e.g., FRAXplus), the AI system can be further extended to preventive care, optimizing the entire process from diagnosis to management (25, 26). Through continuous improvement in data diversity and model interpretability, such frameworks are expected to promote the development of personalized medicine, ultimately enhancing the quality of life for hip fracture patients worldwide.

## 2 Materials and methods

### 2.1 Study subjects

The dataset used in this study consisted of 976 X-ray images of intertrochanteric femoral fractures collected from our hospital and affiliated institutions between June 2020 and October 2025. These X-ray images clearly display intertrochanteric femoral fractures of different grades (A1, A2, A3) according to the 2018 version of the AO/OTA classification.

### 2.2 Dataset preparation

In preparation for input into YOLOv8, the X-ray images underwent a preprocessing stage. This stage included standardizing the images to a uniform size and resolution and converting them to grayscale. Grayscale conversion reduced the number of image channels, improving image processing efficiency.

Under the supervision of an experienced orthopedic surgeon, a medical intern annotated and classified the fracture grades on the X-ray images using the Labelme annotation software. This process constructed a deep learning dataset for intertrochanteric femoral fracture grade detection. The supervision process involved initial training of the intern on AO/OTA criteria, followed by real-time feedback and validation of all annotations by the surgeon. Any discrepancies were resolved via consensus discussions, ensuring adherence to clinical standards. To quantify consistency, a random subset of 100 images was re-annotated by the surgeon, showing high inter-observer agreement (kappa = 0.85). Future work will expand this to include multiple experts for independent annotations to further enhance reliability. Intertrochanteric femoral fractures were classified into three grades: A1, A2, and A3. For clarity, this study adopted a specific naming convention: “A1” represents type A1 intertrochanteric femoral fracture, “A2” represents type A2, and “A3” represents type A3. This naming convention is consistently used in the figures of this paper. In this study, 976 intertrochanteric fracture images were selected for training, including 261 type A1, 579 type A2, and 136 type A3. The aforementioned 976 samples were randomly split into training and test sets in an 8:2 ratio using a random seed method. Although the sample ratios of the three fracture types were severely imbalanced, we employed a class-weighted loss function to mitigate this issue, increasing the loss penalty for misclassification of minority classes. This function assigned higher weights to A1 and A3 fractures during training to reduce bias, but the limited sample size for A3 (136 instances) may still affect subtype performance reliability. Future iterations will incorporate advanced techniques like synthetic data generation to address this imbalance more effectively.

### 2.3 Principles of the YOLOv8 object detection algorithm

The YOLOv8 algorithm inherits and extends the advantages of previous generations in the YOLO series, further enhancing the accuracy and efficiency of object detection. The algorithm introduces several key architectural improvements. First, YOLOv8 adopts CSPDarknet53 as its backbone network. By introducing Cross-Stage Partial (CSP) connections, it enhances information flow, thereby improving feature extraction efficiency and overall network performance. Second, YOLOv8 introduces a Path Aggregation Network (PAN) in the neck structure, enabling the model to effectively fuse features at different scales, particularly excelling when dealing with targets of varying sizes. Compared to previous generations, YOLOv8 abandons the traditional anchor-based strategy and adopts an anchor-free detection approach. This not only simplifies the model architecture and reduces computational burden but also significantly improves the detection accuracy of small targets. In terms of training strategy, YOLOv8 introduces advanced methods such as Mosaic data augmentation and MixUp, and employs a cosine annealing scheduler to optimize the learning rate, significantly improving the model’s generalization ability and convergence speed. These improvements make YOLOv8 perform excellently on multiple object detection benchmarks, showing significant advantages especially in inference speed and detection accuracy. These innovations in YOLOv8 demonstrate great potential and broad application prospects in the field of medical image detection, particularly in fracture detection from medical images, where its high accuracy and efficiency provide strong support for improving diagnostic precision.

## 3 Improved fracture detection algorithm based on YOLOv8

**FIGURE 1:**
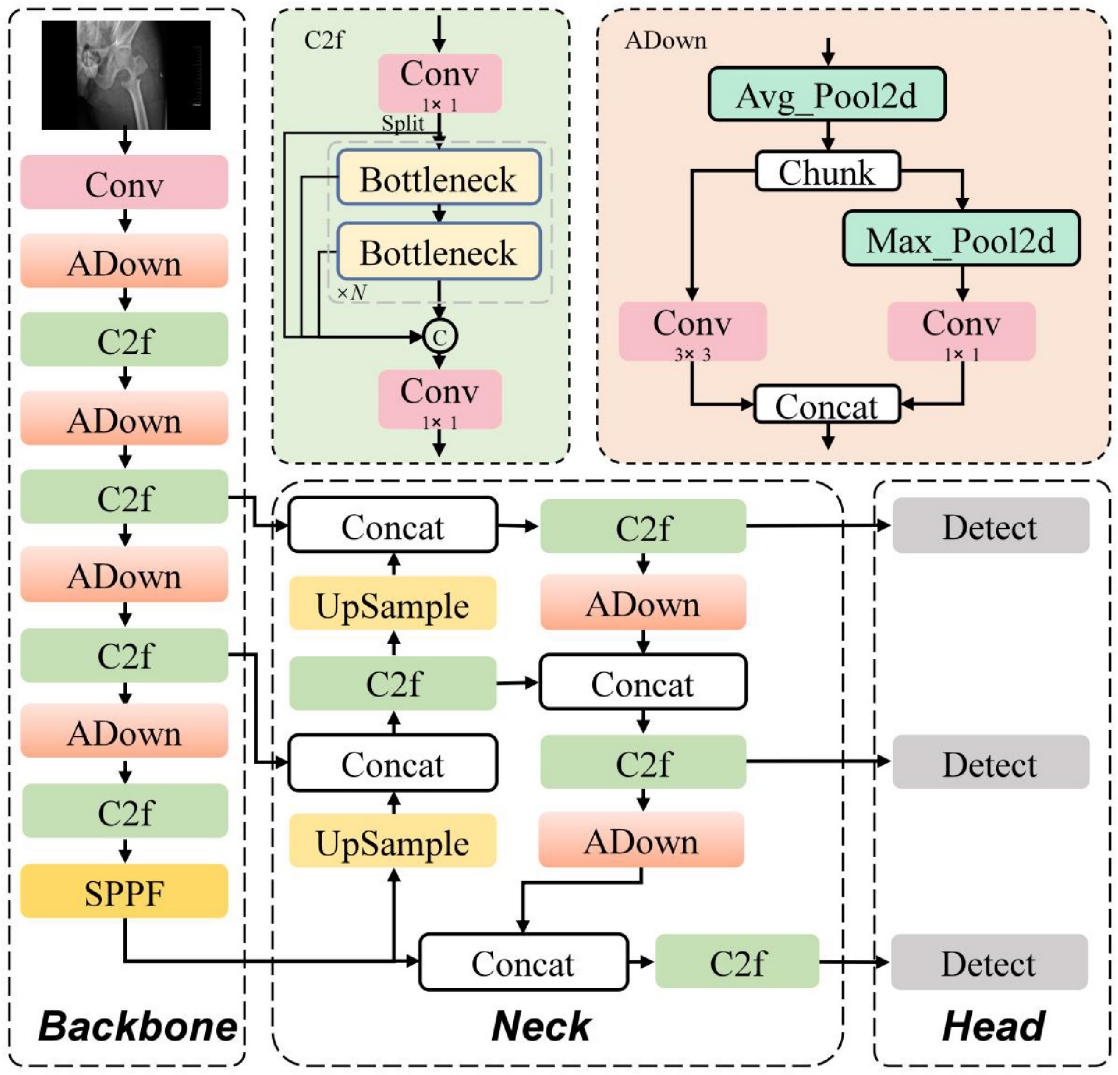
YOLOv8-ADown model architecture.

To improve the localization accuracy and fracture grade judgment accuracy of the model on X-ray images of intertrochanteric femoral fractures, while reducing the model’s computational load and parameters, key improvements were made based on the YOLOv8 framework, proposing the YOLOv8-ADown model. Its structure is shown in Figure 1. The improvement involves replacing the traditional convolutional downsampling (Conv) modules in the backbone and neck networks of YOLOv8 with Average Pooling Downsampling (ADown) modules. This reduces the number of model parameters and computational load, while avoiding the loss of detail during traditional downsampling, thereby enhancing the model’s feature extraction capability for subtle fractures in X-ray images.

### 3.1 Principles of the ADown module

**FIGURE 2:**
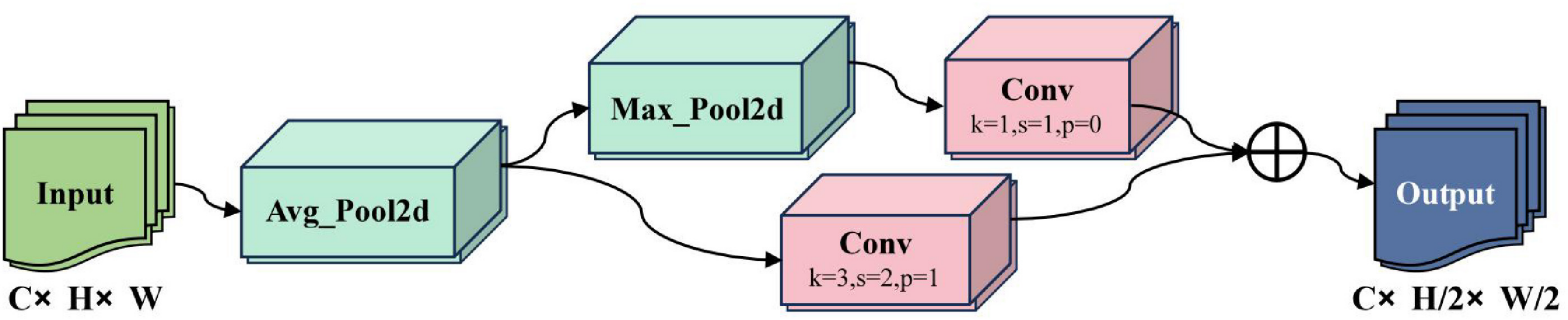
ADown module schematic.

The X-ray image samples in this study contain small fracture targets. The traditional Conv downsampling module performs feature extraction using 3 × 3 convolutions with a stride of 2, which not only increases the number of model parameters and computational load but also fails to effectively capture features of small targets, easily leading to feature loss and missed detections. To address the problem of information loss in small fracture targets, this model introduces the Average Pooling Downsampling (ADown) module into the YOLOv8n model, replacing the Conv downsampling modules in the backbone and neck networks, effectively solving the problem of small fracture target information loss during feature extraction. The workflow of the ADown module is shown in Figure 2.

The ADown module is primarily composed of a 2D Average Pooling layer (AvgPool2d), a 2D Max Pooling layer (MaxPool2d), and a Convolutional layer (Conv). First, the AvgPool2d module performs a 2 × 2 average pooling operation with a stride of 1 on the input feature map, calculating the average value of all pixels in the region. This preserves the main features of the image and removes detailed noise, allowing it to capture finer features and avoid the loss of small target features. Then, the feature map obtained from 2D average pooling is evenly split into two groups along the channel dimension, with each group having half the number of channels. These two groups undergo different downsampling operations. The first group undergoes a 3 × 3 2D Max Pooling layer (MaxPool2d) operation with a stride of 2, halving the feature map size and retaining the maximum value in the region, highlighting features of small targets and suppressing background noise, followed by a 1 × 1 convolution to fuse pixel features. The second group of feature maps undergoes downsampling via a 3 × 3 convolutional layer with a stride of 2, halving the feature map size. Finally, the two processed feature map groups are concatenated to restore the original number of channels.

## 4 Experimental environment and evaluation metrics

### 4.1 Experimental environment

The experiments were conducted on a Windows 11 operating system with an NVIDIA GeForce RTX 3060 Ti 8GB GPU. The virtual environment was configured as follows: Python version 3.9.23, PyTorch version 2.0.0, and CUDA version 11.8.

This study utilized the YOLOv8 deep learning network and proposed a new YOLOv8-ADown fracture detection method. The official Ultralytics YOLOv8n model implementation (version 8.2.103) was used. The initial learning rate (Lr0) was set to 0.01, the final learning rate (Lrf) to 0.01, batch size to 16, and the number of training epochs to 200. Image augmentation techniques (e.g., Mosaic augmentation, horizontal flip, scale, and translate) were used to enhance model robustness. The SGD optimizer with a weight decay of 5 × 10^–4^ was employed.

### 4.2 Evaluation metrics

This experiment used Precision (P), Recall (R), and mean Average Precision (mAP) to evaluate the performance of the YOLOv8-ADown model in detecting fractures.

Here, Precision *P* represents the proportion of actual positive samples among the predicted positive samples, calculated as:


$$P=\frac{T\,P}{T\,P+F\,P}$$

Where: *TP* (True Positive) is the number of correctly classified positive samples, i.e., targets labeled as fracture correctly detected as fracture; *FP* (False Positive) is the number of incorrectly detected negative samples, i.e., targets not labeled as fracture incorrectly detected as fracture.

Recall *R* is calculated as follows:


$$P=\frac{T\,P}{T\,P+F\,N}$$

Where: *FN* (False Negative) is the number of missed positive samples, i.e., targets labeled as fracture that were not detected.

The mean Average Precision *mAP* uses *mAP*_50_ (*IoU* threshold of 0.5) and *mAP*_50:95_ (*IoU* threshold from 0.5 to 0.95 in steps of 0.05) as evaluation metrics. The *mAP* is calculated as follows:


$$m\,A\,P=\frac{1}{N}\,\overset{N}{\underset{i=1}{\sum }}A\,P_{i}$$

Where: *N* is the number of target classes. In this study, *N* = 1, *AP*_*i*_ is the Average Precision for class *i*, calculated as:


$$A\,P_{i}=\int_{0}^{1}P_{i}\cdot R_{i}$$

Where: *P*_*i*_ and *R*_*i*_ represent the precision and recall for detection class *i*, respectively.

## 5 Results analysis

The Precision (P), Recall (R), mAP50, and mAP50:95 of the original YOLOv8 and the improved YOLOv8-ADown model on the dataset used in this study are shown in Table 1. The original YOLOv8 model achieved an overall mAP50 of 80.5% for these fracture types, while the improved YOLOv8-ADown model increased the overall mAP50 to 81.7%. This performance is comparable to Yang et al. (27), who reported an mAP50-95 of 85.9% for vertebral fracture classification using YOLOv8-Seg, highlighting the versatility of YOLOv8 in fracture detection. This indicates that the improved model enhanced the performance in fracture detection. Specifically, the detection precision (P) of the improved model for A1, A2, and A3 type fractures increased by 7.3, 3.5, and 7.8%, respectively, compared to the original model, effectively improving the detection precision for different fracture types.

**TABLE 1:** Comparison of model accuracy and parameters before and after improvement.

Model	Class	All	A1	A2	A3
YOLOv8	Images	196	50	109	37
	Instances	196	50	109	37
Params	Box(P)	0.766	0.723	0.782	0.794
3.01M	R	0.714	0.66	0.86	0.622
FLOPs	mAP50	0.805	0.772	0.867	0.776
8.1G	mAP50-95	0.615	0.599	0.677	0.565
YOLOv8-ADown	Images	196	50	109	37
	Instances	196	50	109	37
Params	Box(P)	0.828	0.796	0.817	0.872
2.64M	R	0.757	0.732	0.89	0.649
FLOPs	mAP50	0.817	0.773	0.868	0.81
7.3G	mAP50-95	0.625	0.603	0.685	0.591

Furthermore, the improved YOLOv8-ADown model reduced the number of parameters (Params) by 12.3% and the computational complexity (FLOPs) by 9.8% compared to the original YOLOv8 model. YOLOv8-ADown significantly reduced the model’s parameters and computations, decreasing computational demands, making it more suitable for deployment on edge devices with limited computing power, and potentially improving detection speed under the same computational constraints.

**FIGURE 3:**
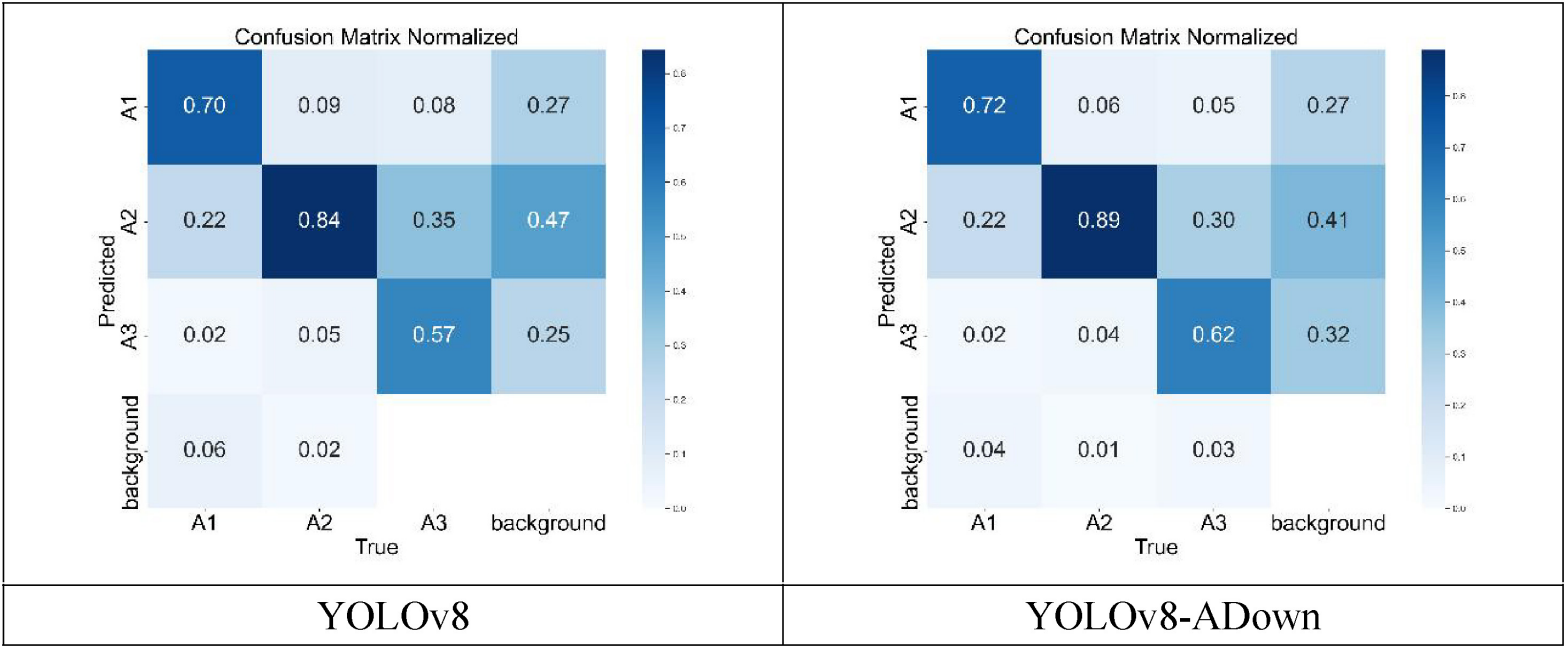
Comparison of normalized confusion matrices of the models.

Figure 3 details the recognition results for the three fracture types. The performance of the YOLOv8 and the improved YOLOv8-ADown algorithms was evaluated on the dataset of 976 fracture samples constructed in this study. Among the 261 analyzed A1-type fractures, the YOLOv8 model correctly identified 70%, while the YOLOv8-ADown model correctly identified 72%, indicating that accuracy in detecting A1-type fractures still needs improvement. Similarly, among the 579 examined A2-type fractures, the YOLOv8 model accurately identified 84%, while the improved model further increased this to 89%, demonstrating the algorithm’s effectiveness in identifying A2-type fractures. Notably, among the 136 A3-type fractures in the dataset, only 57% were correctly identified by YOLOv8, while the improved YOLOv8-ADown achieved a correct identification rate of 62%. The detection accuracy for A3-type fractures was the lowest, which may be related to the smaller number of A3-type fracture samples in the dataset.

**FIGURE 4:**
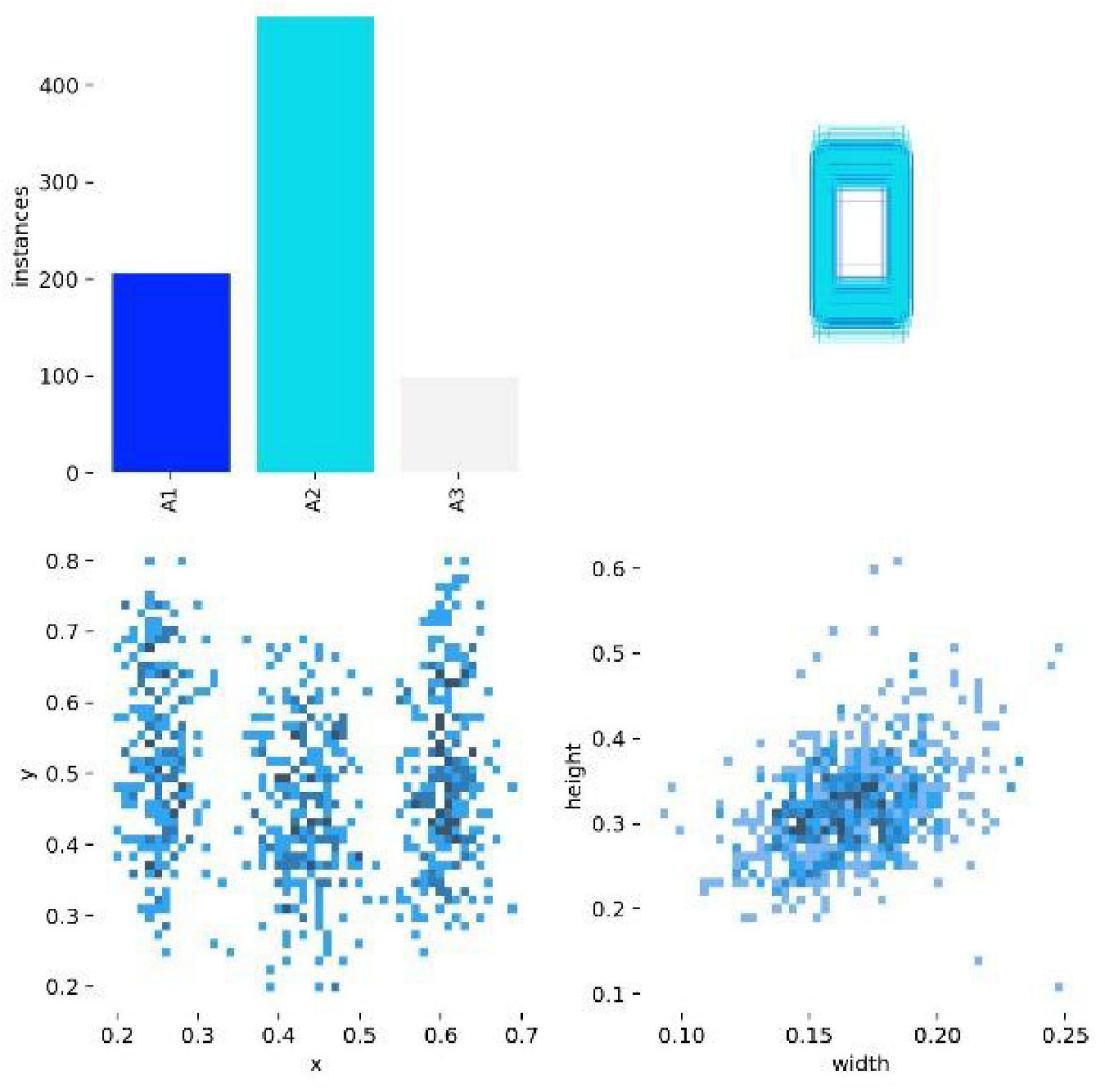
Distribution and attribute analysis of training set data.

Figure 4 shows the data distribution of the training set, including the number of instances per class, the size and quantity of bounding boxes, the location of center points relative to the entire image, and the aspect ratio of targets in the images. The top-left corner shows the instance count for each class in the training set, i.e., the total number of occurrences of that class across all training images. It can be seen that the sample sizes for A1, A2, and A3 type fractures are uneven, providing a basis for understanding the sample size of each class and guidance for data augmentation. The top-right corner shows the effect of aligning and overlaying the bounding boxes of all classes centered on the image origin, allowing for an intuitive understanding of the shape, size, and approximate positional distribution of the bounding boxes for each class. The bottom-left corner shows the distribution of target center points relative to the entire image, allowing further analysis of the spatial location preference of the targets or defects within the image. The bottom-right corner shows the distribution of the height-to-width ratios of the target bounding boxes in the images, allowing further analysis of the size and shape preferences of the defect bounding boxes.

**FIGURE 5:**
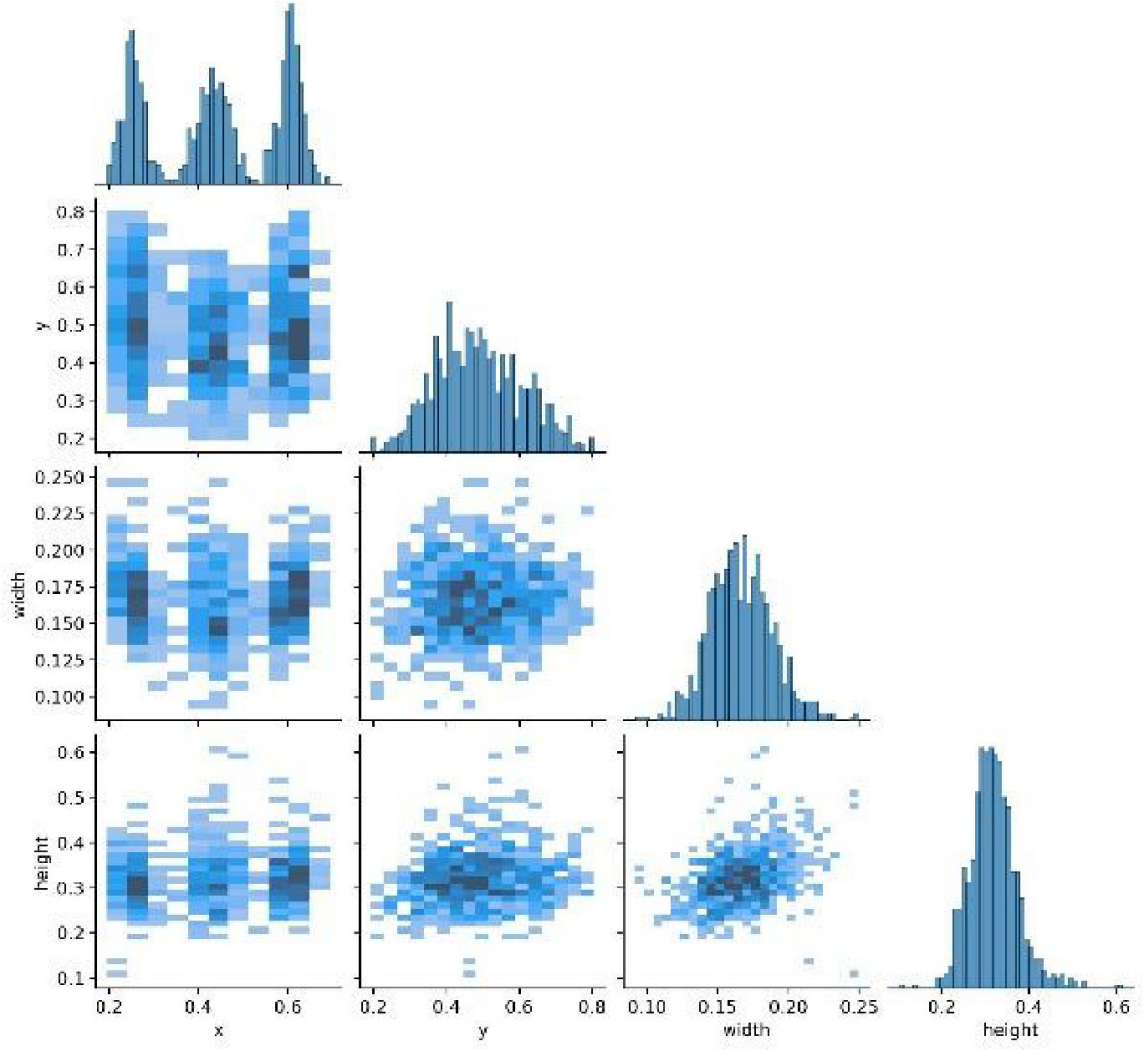
Correlation matrix of bounding box attributes.

Figure 5 depicts the correlation matrix (pairs plot) between attributes of the annotation boxes (labels), such as the normalized horizontal and vertical coordinates of the center point (x, y) and the normalized width and height. The diagonal of the matrix shows the one-dimensional distribution histograms for each attribute. The off-diagonal plots are two-dimensional scatter plots between two different attributes, showing their relationships; darker colors indicate denser data points.

**FIGURE 6:**
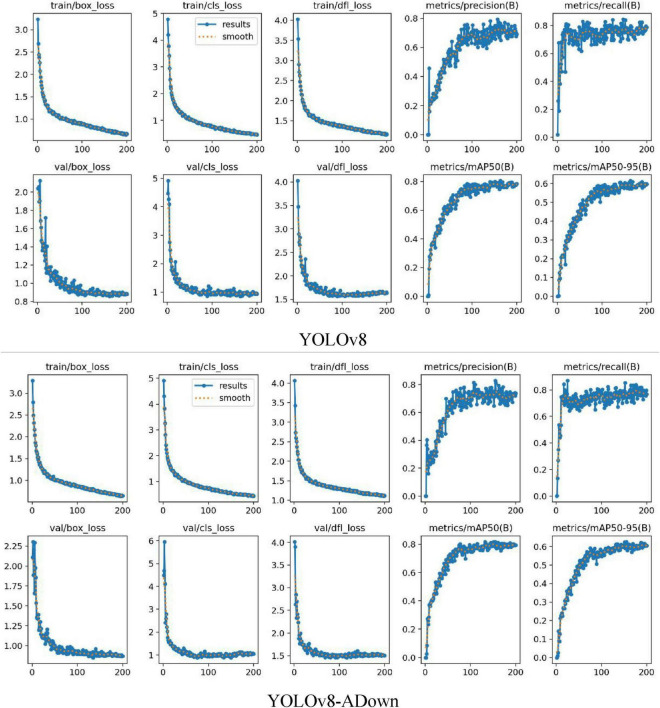
Comparison of model loss and accuracy curves.

Figure 6 shows the curves of loss changes and performance metrics (Precision, Recall, etc.) during model training. Figure 6 shows the loss curves (downward trend) and performance metrics (upward trend). The model uses loss functions to evaluate the difference between predicted and true values; these loss functions significantly impact model performance as they guide the training process toward more accurate predictions. The loss functions are divided into classification and regression parts: The classification loss (cls_loss) uses Binary Cross-Entropy Loss to calculate the difference between predicted and true classes. A lower classification loss indicates more precise classification of detected objects into their respective categories. The regression branch losses include dfl_loss (Distributed Focal Loss) and box_loss (Bounding Box Loss). The box_loss calculates the loss for the predicted bounding box’s position and size relative to the ground truth box, using the Intersection over Union (IoU) metric. A higher IoU value indicates higher localization accuracy, reflecting a more precise overlap between the predicted and ground truth bounding boxes. In summary, these loss functions are indispensable for optimizing the YOLOv8 model, thereby driving improvements in detection and classification accuracy.

The curves on the right side of Figure 6, showing an upward trend, represent the changes in Precision, Recall, mAP50, and mAP50:95 during training. Precision is defined as a measure of the accuracy of the model’s positive predictions, calculated as the ratio of correctly identified positive instances (True Positives) to all instances identified as positive (True Positives + False Positives). Higher precision indicates that the model makes fewer false positive errors. Recall represents the proportion of actual positive samples correctly identified by the model, essentially measuring the model’s ability to identify all relevant instances in the dataset. It is calculated as the ratio of True Positives to the sum of True Positives and False Negatives. mAP50 and mAP50:95 represent the mean Average Precision at an IoU threshold of 0.5 and the average of mean Average Precision computed at IoU thresholds from 0.5 to 0.95 with a step size of 0.05, respectively, reflecting the model’s true detection performance from different dimensions.

**FIGURE 7:**
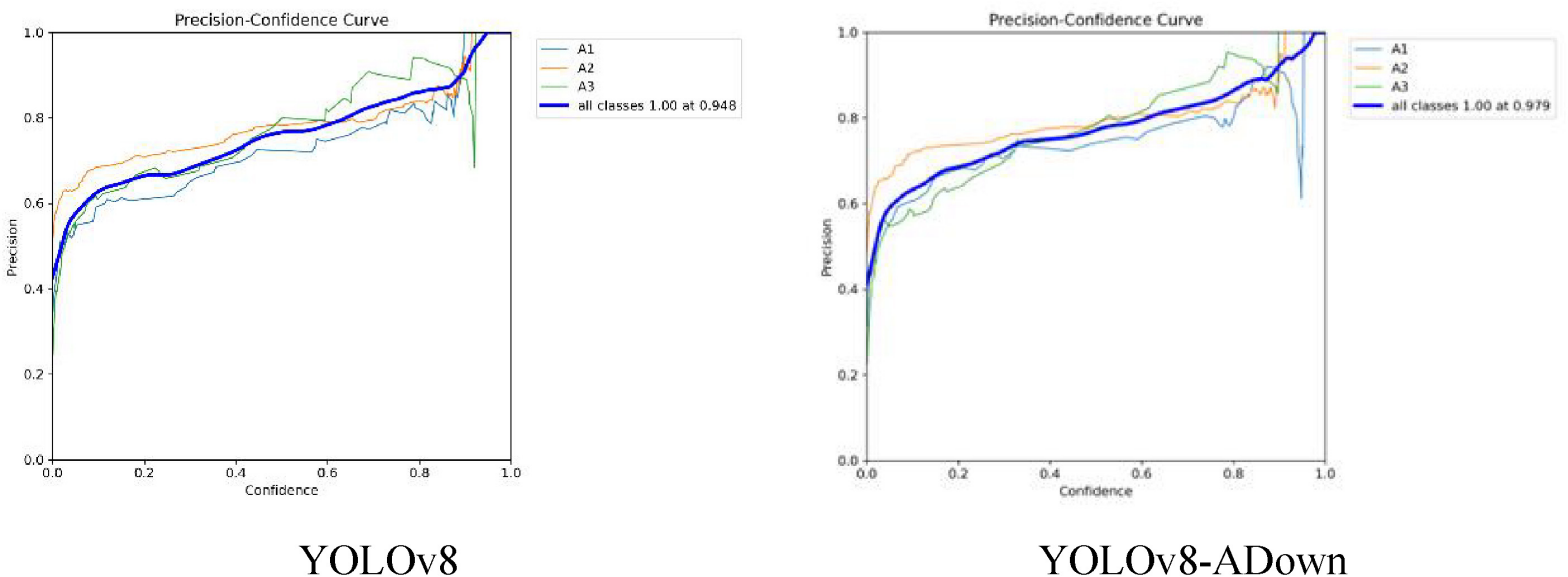
Comparison of model P curves.

Subsequently, a comparative analysis of the YOLOv8 model before and after improvement was conducted using four key metrics—Precision, Recall, F1-Score, and Average Precision (AP)—to provide a comprehensive evaluation of its performance enhancements across various dimensions. First, the Precision for the three fracture types was examined, which in this experiment refers to the proportion of correctly predicted fracture types out of the total number of fracture types predicted by the model. As shown in Figure 7, which compares the Precision curves of the models before and after improvement, the enhanced YOLOv8-ADown model achieved a Precision of 0.979 for recognizing all fracture classes, representing an increase of 0.031 compared to the original YOLOv8’s Precision of 0.948. This result clearly demonstrates that the improved YOLOv8-ADown model exhibits a significant enhancement in detection accuracy relative to the original model.

**FIGURE 8:**
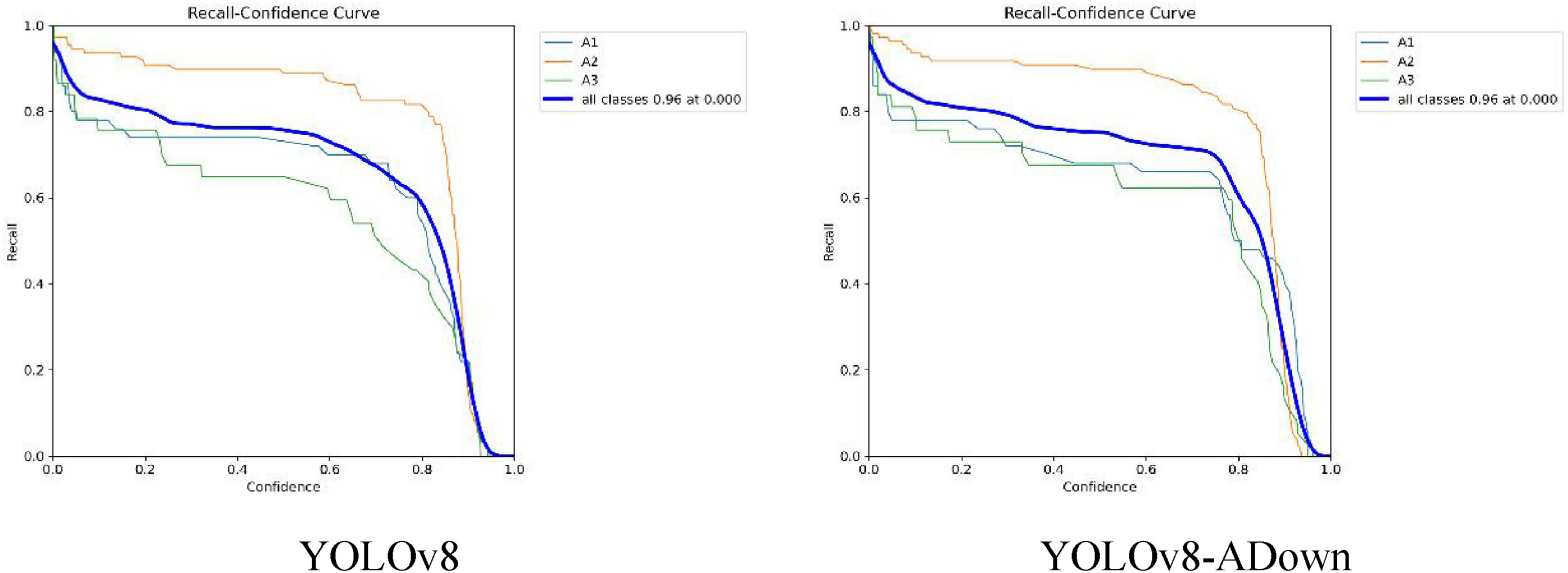
Comparison of model R curves.

Recall (R) represents the proportion of correctly predicted fracture types out of the total number of actual fracture types. Figure 8 shows the comparison of the R curves between the original and improved models. Although the comprehensive recall score in the legend is 0.96 for both, showing no obvious overall improvement, the recall curve for each individual fracture type in the improved model shows some enhancement compared to the pre-improvement model, indicating that the improved YOLOv8-ADown model has a certain positive effect on recall.

**FIGURE 9:**
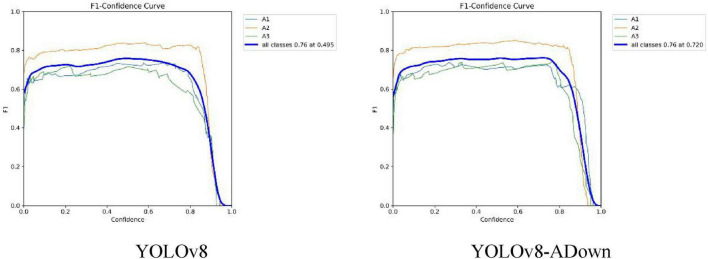
Comparison of model F1 curves.

The F1-Score is the harmonic mean of Precision and Recall, used to comprehensively evaluate the performance of a binary classification model. Setting the confidence threshold to 0.5, Figure 9 shows the F1 curve comparison between the original and improved models. It can be observed that the F1 scores for all three fracture types (A1, A2, A3) slightly improved. Although the comprehensive F1 score did not show a significant increase, the confidence threshold corresponding to the highest F1 score increased from 0.495 to 0.720. This indicates that the model achieves a better F1 score at higher confidence thresholds, making the model’s predictions more reliable.

**FIGURE 10:**
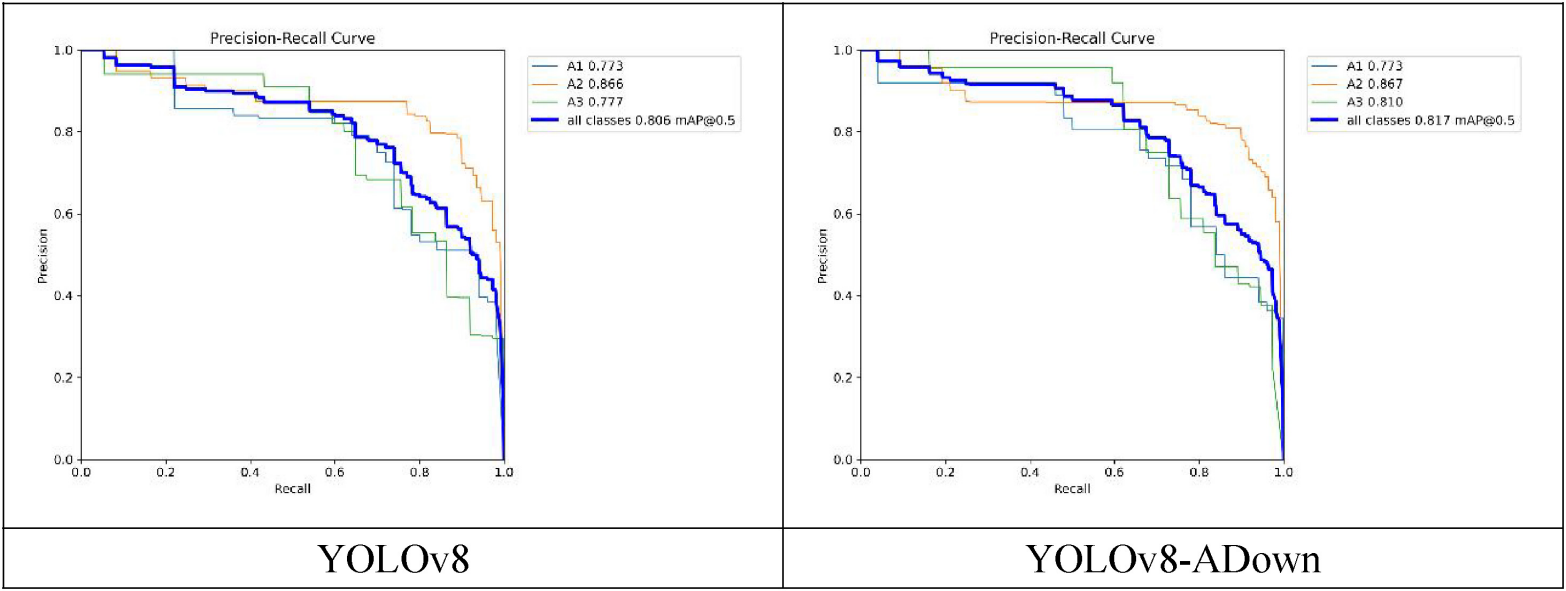
Comparison of model PR curves.

As the most important among the four metrics, the Average Precision (AP) value provides a more comprehensive measure of the relationship between Precision and Recall. Essentially, the AP value is the area under the Precision-Recall (P-R) curve; a larger area indicates a higher AP value, meaning better detection accuracy for that class of objects. Figure 10 shows the comparison of the P-R curves between the original and improved models. After replacing the traditional Conv modules with the ADown downsampling modules, the changes in AP values for each fracture type can be seen. Among them, the AP value for A1-type fractures showed no significant change, but the P-R curve became noticeably smoother. The AP value for A2-type fractures increased from 0.866 to 0.867. The AP value for A3-type fractures improved more substantially, increasing from 0.777 to 0.810, a gain of 0.033. Overall, the mean Average Precision (mAP) for the three fracture types increased by 0.011, from 0.806 to 0.817. This first comparative experiment confirms that the introduction of the ADown downsampling module leads to a relatively clear improvement in the model’s detection performance.

**FIGURE 11:**
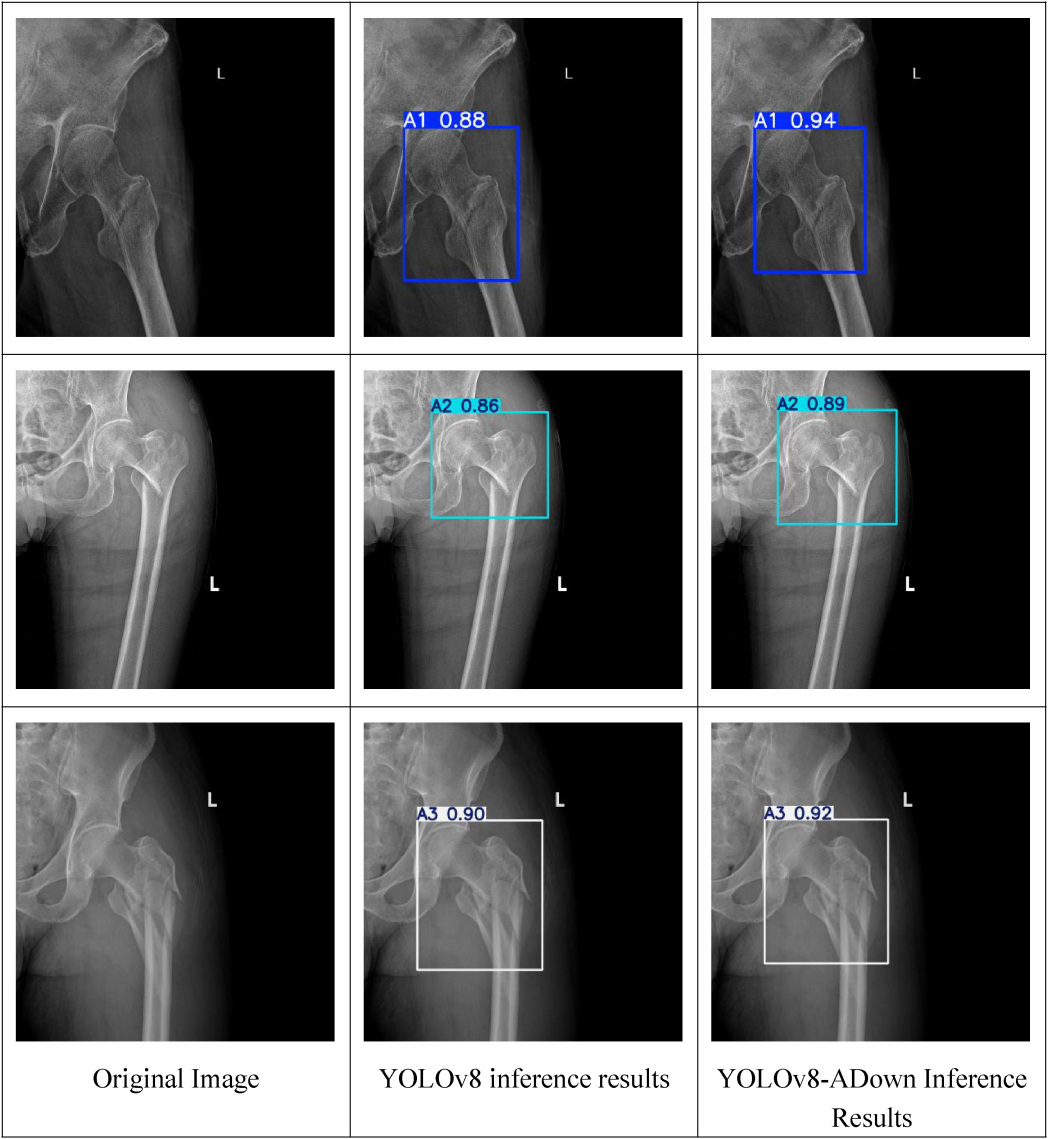
Visualization comparison of model recognition results.

Figure 11 shows the visual comparison of the recognition results between the YOLOv8 model and the YOLOv8-ADown model. It can be observed that the improved model increased the recognition confidence for all three fracture types. The confidence for A1-type fractures increased from 0.88 to 0.94, for A2-type from 0.86 to 0.89, and for A3-type from 0.90 to 0.92. This further proves that the improved model enhances the recognition capability for the three fracture types.

Since this study only collected 976 original images, there is an issue of limited data volume. To further validate the robustness of the model, data augmentation was performed on the original images and labels. Various data augmentation methods are available; this study adopted horizontal flipping to simulate the left-right symmetric structure of the human body, enabling a single image to serve for predicting both left and right fracture types. Additionally, the built-in data augmentation methods of the YOLOv8 model were employed during training, including hue adjustment (hsv_h), saturation adjustment (hsv_s), brightness adjustment (hsv_v), translation, scaling, etc., to further test the model’s robustness in training.

Experiments were conducted on the augmented dataset, with a random 7:3 split for training and validation sets. The training results are shown in Table 2. The improved YOLOv8-ADown model achieved improvements of 6.1, 2.6, 1.1, and 1.2% in the four parameters P, R, mAP50, and mAP50-95 on the augmented dataset, respectively. The detection accuracy of the model on the augmented dataset remained at a good level, demonstrating good robustness.

**TABLE 2:** Training results of the models before and after improvement on the augmented dataset.

Model	Class	All	A1	A2	A3
YOLOv8	Images	586	176	298	112
	Instances	586	176	298	112
	Box(P)	0.748	0.715	0.782	0.781
	R	0.706	0.642	0.86	0.613
	mAP50	0.794	0.758	0.867	0.767
	mAP50-95	0.601	0.592	0.677	0.556
YOLOv8-Adown	Images	586	176	298	112
	Instances	586	176	298	112
	Box(P)	0.809	0.789	0.806	0.864
	R	0.732	0.723	0.879	0.64
	mAP50	0.805	0.759	0.854	0.798
	mAP50-95	0.613	0.601	0.672	0.578

**FIGURE 12:**
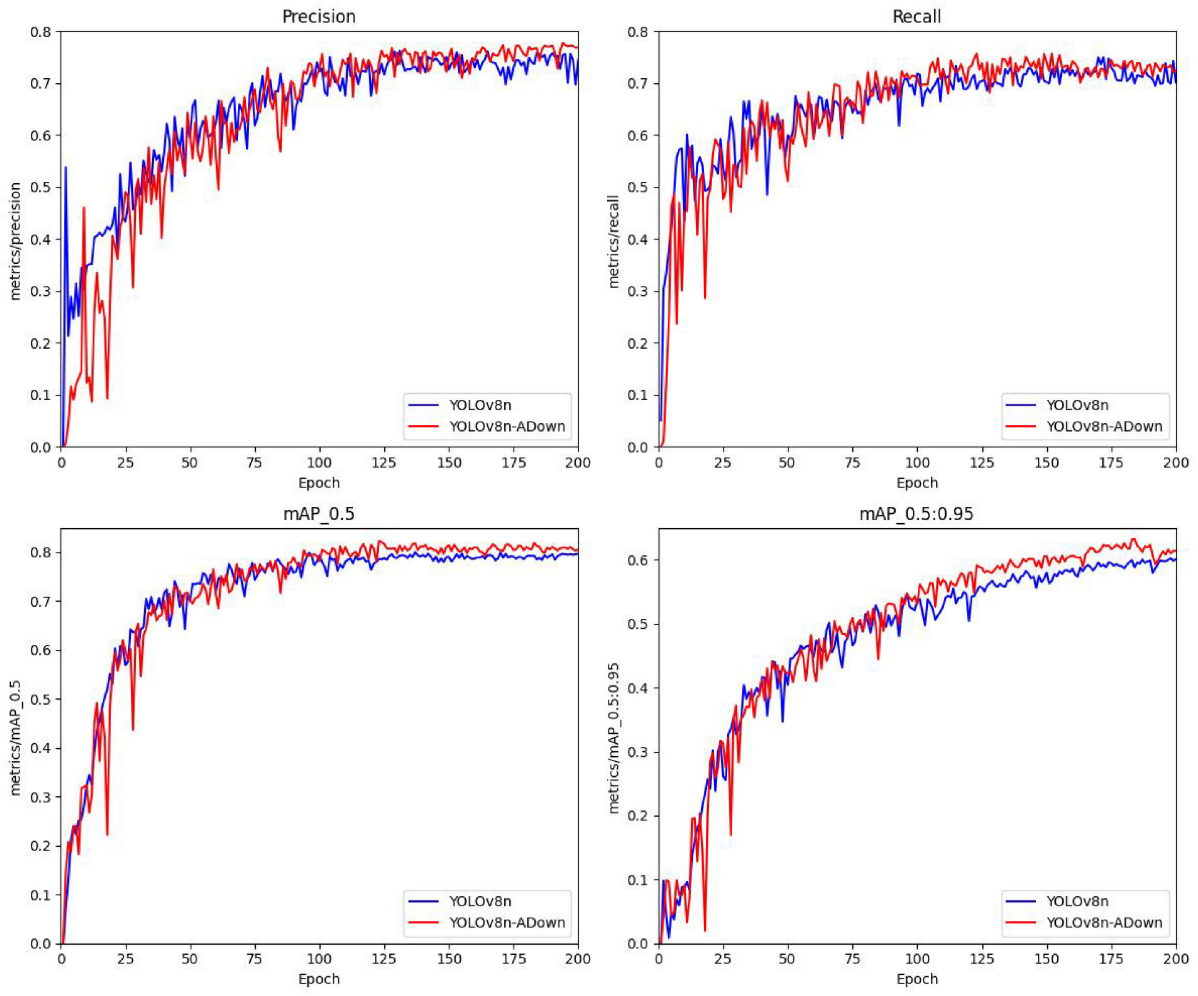
Comparison of accuracy curves between the improved and original models on the data augmented dataset.

The core focus before and after improvement lies in the changes of the four parameters P, R, mAP50, and mAP50-95. Therefore, to more intuitively display the changes in model accuracy before and after improvement, a comparison curve of the training accuracy before and after improvement on the augmented dataset was plotted, as shown in Figure 12.

## 6 Discussion

### 6.1 Research summary and main contributions

This study introduced YOLOv8-ADown, an enhanced framework for intertrochanteric femoral fracture detection. Key improvements, such as replacing convolutional downsampling with ADown modules, boosted feature extraction for small targets, yielding an overall mAP50 of 81.7% (vs. 80.5% for baseline YOLOv8). Precision gains for A1, A2, and A3 fractures were 7.3, 3.5, and 7.8%, respectively, while parameters and FLOPs decreased by 12.3 and 9.8%. These outcomes highlight our model’s efficiency-edge device potential, aligning with lightweight trends like GSCDown in transmission line detection (28).

The innovation of this work lies in being the first to achieve fine-grained classification and localization of A1-A3 type fractures according to the AO/OTA classification, breaking through the limitations of traditional binary classification models. Wang et al. (29) applied an enhanced YOLOv8 for distal radius fracture classification, achieving high accuracy in AO typing, which aligns with our focus on computational efficiency. Furthermore, by combining attention mechanisms and pooling operations, the model’s feature extraction capability in complex backgrounds is enhanced. This is consistent with the Local Importance-based Pooling (LIP) method proposed by Gao et al. (30), which optimizes the downsampling process through adaptive weights to improve discriminative feature retention.

### 6.2 Research limitations and shortcomings

Although the YOLOv8-ADown model performs excellently on multiple metrics, the following limitations remain:

First, the data sample imbalance issue is prominent. With only 136 samples for A3-type fractures, its recognition rate is relatively low (62%). To enhance the credibility of subtype-specific claims, we recommend that future studies prioritize data balancing through multi-center collaborations or oversampling methods. This will ensure more equitable representation of all fracture classes and improve model generalizability. The reliability of the new classification system is affected by the sample size, and the generalization ability for minority categories is insufficient (31). Similarly, biomechanical analyses reveal that the management of the fracture line requires caution; for instance, interfragmentary compression may impose additional stress on the physeal plate, suggesting that AI models require diverse, multi-center data to mitigate such clinical decision-making biases (32). Similar challenges were noted by Sun et al. (33) in predicting post-surgical pain, where sample size limitations impacted model generalizability, underscoring the need for expanded datasets in orthopedic AI studies.

Second, the single-center retrospective data may introduce biases from equipment differences and imaging parameters, limiting the model’s generalizability. To mitigate this, future studies should involve multi-center prospective data collection, encompassing varied imaging protocols and demographic populations. This approach will help validate the model’s performance across diverse clinical settings and enhance its adoption in real-world scenarios. The performance of AI-assisted systems may fluctuate in multi-center validations; for example, while sensitivity in pelvic fracture detection might improve, specificity could decrease, highlighting the necessity of external validation (34).

Third, the annotation process may introduce subjectivity. Although annotations were performed by a medical intern under rigorous supervision, we acknowledge that involving multiple specialists could reduce potential bias. In future studies, we plan to adopt a dual-annotation framework with blinded reviews to improve generalizability and minimize subjectivity in label generation.

Additionally, the model’s ability to handle complex fractures (such as comminuted A3-type fractures) is insufficient. Although deep learning models for proximal femoral fracture detection may outperform radiologists, their error rates increase in cases with skeletal abnormalities, reflecting the model’s poor adaptability to morphological variations (35).

### 6.3 Future research directions

Addressing the above limitations, future research can focus on the following aspects: First, expanding the dataset and conducting multi-center validation. Drawing on global hip fracture epidemiology studies (36), integrating diverse data across ages, geographies, and clinical settings can enhance model robustness. Second, the model architecture can be further optimized. For instance, incorporating self-attention mechanisms like Transformers, or methods like using deformable convolutions to improve YOLOv8 (37), can enhance the detection capability for irregularly shaped targets. Simultaneously, multi-modal data fusion is an important direction. Digital twin technology, by combining multi-modal images such as CT and MRI, enables personalized anatomical modeling (38). Simultaneously, the integration of material science data, such as the biomechanical performance and fatigue behavior of different screw materials, represents a crucial direction for extending the AI system into an implant recommendation platform for end-to-end patient management (39). Our model could be extended to 3D fracture detection and risk prediction. For instance, Gao et al. (40) demonstrated the utility of YOLOv8-seg in classifying intervertebral disc anomalies from CT images, suggesting potential for adapting our model to other spinal disorders. Furthermore, clinical deployment requires enhanced interpretability. The multimodal large language model (MLLM) by Nam et al. (41) improves diagnostic transparency through visual question answering. Our work could introduce similar techniques like Grad-CAM++ to generate heatmaps. Finally, cross-disease generalization research has potential. Multi-scale optimization studies on YOLOv8 indicate that the model can be adapted to other fracture types (e.g., femoral neck fractures) (42). Future work could explore its feasibility as a universal auxiliary platform.

## 7 Conclusion

The YOLOv8-ADown model, improved through average pooling downsampling, achieves a balance between accuracy and efficiency in the detection of intertrochanteric femoral fractures, providing an efficient and lightweight AI-assisted tool for clinical practice. In the future, through multi-center data validation, architectural innovation, and clinical integration, it is expected to promote the practical application of AI in orthopedic imaging, ultimately optimizing the allocation of medical resources and improving patient outcomes. Compared to existing research, the advantages of this framework in fine-grained classification and computational efficiency lay the foundation for personalized medicine in fracture diagnosis.

## Data Availability

The original contributions presented in the study are included in the article/supplementary material, further inquiries can be directed to the corresponding authors.
